# Ten Practical Tips for a Successful Presentation at an Infectious Disease Clinical Case Conference

**DOI:** 10.1093/ofid/ofaf387

**Published:** 2025-07-01

**Authors:** Takahiro Matsuo, Pablo C Okhuysen, Ben J Barnett

**Affiliations:** Department of Infectious Diseases, Infection Control and Employee Health, The University of Texas MD Anderson Cancer Center, Houston, Texas, USA; Division of Infectious Diseases, Department of Medicine, The University of Texas Health Science Center at Houston, Houston, Texas, USA; Department of Infectious Diseases, Infection Control and Employee Health, The University of Texas MD Anderson Cancer Center, Houston, Texas, USA; Division of Infectious Diseases, Department of Medicine, The University of Texas Health Science Center at Houston, Houston, Texas, USA

**Keywords:** conference, infectious diseases, medical education, presentation

## Abstract

This mini-review provides 10 practical tips for infectious disease trainees presenting at clinical case conferences. These tips, grounded in principles from education theory, aim to enhance the effectiveness of case presentations.

## Teaser

This review offers ten practical tips, grounded in educational theory, for Infectious Disease (ID) trainees presenting at ID Clinical Case Conferences. These tips provide a roadmap to help trainees navigate the challenges of case presentations, enhancing both the quality of presentations and the learning experience for their audience.

## Graphical Abstract

**Graphical Abstract:**
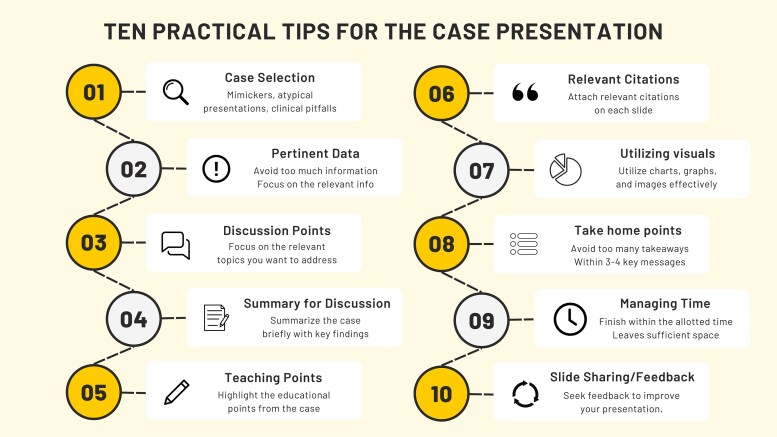

Clinical case conferences have been a long-standing tradition in medical education and essential to patient care–related educational activities [1]. These conferences offer learning opportunities for presenting trainees and their audience, focusing on enhancing clinical reasoning in diagnostic and management topics [2]. The case presentation process helps trainees acquire medical knowledge applicable to patient care while teaching them to review literature, focus discussions, and present effectively. Peer trainees also benefit by learning from diverse cases, including those that they may not have encountered.

Historically, as in other specialties, case conferences have been a standard educational component of infectious disease (ID) training, with participation required by the Accreditation Council for Graduate Medical Education as part of fellowship programs [3]. Their effectiveness depends on the presenters' ability to deliver their presentations and engage the audience. While case conference presentations are ubiquitous in training programs, opportunities for structured education for delivering effective case conference presentations vary by program, with some institutions lacking formal training or feedback opportunities, leaving trainees to develop skills solely by observing senior or peer colleagues at past conferences. Consequently, some trainees might struggle to deliver effective presentations.

ID case conferences are unique in their emphasis on diagnostic uncertainty and complex clinical reasoning, requiring integration of host factors, epidemiologic context, microbiologic factors, and therapeutic considerations (Table 1). While other resources have addressed general case presentation skills [2, 4, 5], few have addressed the unique considerations and teaching opportunities specific to the ID context. In this article, we define “ID clinical case conference” as a structured, trainee-led session, typically 30 to 60 minutes in length, featuring a case presentation followed by a focused teaching segment and attended by a diverse multilevel audience including trainees and faculty from ID, pharmacy, microbiology, and related fields. While this format is widely used, we acknowledge that alternative structures—such as faculty-moderated discussions, multispecialty conferences (eg, with radiology, surgery, pathology, or other specialties), structured didactics, and case-based journal clubs—may serve important educational goals depending on the institutional context. This mini-review addresses this challenge by offering 10 practical tips to help trainees prepare and deliver successful presentations, grounded in principles from education theory.

**Table 1.** Unique Core Domains in Infectious Disease Case Conferences

Core Domain	Key Elements	Implications for Case Presentation
Host factors	Immune status (eg, HIV, cancer, neutropenia/lymphopenia, transplant, corticosteroids, chemotherapy), comorbidities (eg, diabetes, CKD, malignancy, cirrhosis), anatomic risk (eg, prostheses, devices, obstructions), behavioral/social risks (eg, IVDU, sexual activity, hygiene), functional status, colonization history	Provide a concise, clinically focused host summary
Epidemiologic context	Local/endemic infections, outbreaks, seasonality, environmental exposures (soil, water, ventilation systems), nosocomial vs community acquired	Structure exposure history clearly and integrate relevant epidemiology
Microbiologic factors	Common site-specific pathogen patterns, resistance profile, virulence mechanisms (eg, biofilm), coinfections	Present microbiologic findings clearly and explain diagnostic relevance and how they guided clinical decisions
Therapeutic considerations	Antimicrobial spectrum and bioavailability, PK/PD (tissue penetration), breakthrough infections, optimal dosing, adverse effects, and source control (eg, surgery, drainage, device removal)	Address treatment strategy, including antimicrobial management, source control, and stewardship considerations

Abbreviations: CKD, chronic kidney disease; IVDU, intravenous drug use; PK/PD, pharmacokinetics/pharmacodynamics.

## TEN TIPS FOR EFFECTIVE ID CASE PRESENTATION

### Choose Educationally Relevant Cases and Topics

A common challenge for trainees is deciding which case to present [4, 5]. A helpful approach is determining if the presenter can summarize the case in 1 sentence that delivers the key lesson for the audience. One framework for categorizing cases for large-group educational purposes is to decide if the focus of the presentation should be on the diagnostic or management aspects of the case [2]. While some trainees believe that only rare or esoteric cases are appropriate to present, any case with a strong educational message can be suitable. Multiple factors likely contribute to trainees' tendency to prioritize unusual or rare cases. This includes the misconception that common diagnoses will not interest an audience largely composed of experienced faculty members or that the primary purpose of case presentations is to transmit novel information. Implicit or explicit messages from faculty or training programs may also contribute to the belief that the depth of a literature review is the primary measure of presentation quality rather than the clarity and educational structure of the case itself. Rather than focusing on whether a case is rare or complex, presenters should prioritize clarity of purpose by selecting cases that illustrate a specific learning objective. The educational impact of a case depends less on its novelty and more on how effectively it is structured to promote audience engagement and reflection. An intentional and goal-oriented approach to case selection allows presenters to better align content with the learning needs of their audience. Effective options include atypical presentations of common diseases [5], uncommon but important conditions for ID physicians to recognize, cases with diverse management approaches, or noninfectious mimickers due to malignancy or autoimmune disease. When presenting a rare disease, presenters should discuss when to suspect it, and they should compare it with more common diseases with similar presentations. It is also important to ensure that the same topic has not been recently presented. New trainees may benefit discussing potential case topics with senior fellows or faculty mentors for advice and historical perspective. Keeping a database indexed by presentation date and diagnosis helps prevent content duplication.

### Include Pertinent Positives and Negatives

While it is essential to include relevant information that makes the case realistic, there is no need to include every detail of the patient's information unrelated to the discussion, especially since case presentations are time bound [5]. Furthermore, understanding the audience is crucial; for instance, when an ID group is the audience, basic information may be omitted, whereas presentations to other specialties or medicine residents may require more foundational details to ensure clarity. A typical presentation starts with the chief concern or reason for consultation, followed by the history of present illness, a review of systems, a medical and social history, and objective data (eg, vital signs, physical findings, laboratory test results, and imaging). However, the presenter can adjust the sequence and priorities based on the case and intended teaching points. For example, if the patient has a complex medical background and treatment history, it may be more effective to start with the current reason for consultation and focus on key information related to the current issue, omitting unnecessary details about the clinical course. This approach helps the audience focus on collecting relevant information related to the main discussion. With this in mind, it is helpful to include not only pertinent positive findings in the case but key negative or absent findings that will allow the audience to develop a prioritized differential diagnosis or management strategy. In cases involving prolonged hospitalizations or multiple admissions, a succinct timeline graphic or bullet-point summary can be especially helpful to clearly convey the clinical course, avoiding confusion that may arise from a lengthy narrative format. Letting the audience ask questions after presentation of the case can allow time for details and clarifications, if needed. However, caution must be taken to avoid needless time consumption and diversions from the educational points intended.

To guide information selection, presenters may benefit from applying cognitive load theory [6]. This framework categorizes mental effort during learning into 3 types: *intrinsic load*, which reflects the inherent complexity of the case; *germane load*, which is the mental effort directed toward understanding and applying clinical reasoning; and *extraneous load*, which arises from irrelevant information or presentation choices that do not support learning. Presenters should aim to preserve intrinsic load, enhance germane load through clear structure and reasoning process, and minimize extraneous load by excluding distracting or unnecessary details. Because the same clinical data may represent intrinsic, germane, or extraneous load depending on the format and educational goal of the session, content should be tailored to the audience and aligned with the case's teaching objective.

### Consider the Points for Discussion

In most ID case conferences, presenters are expected to include 1 or more interactive pauses during the presentation to invite audience participation. These discussions typically involve the audience sharing its clinical thinking process, often guided by the presenter through targeted questions. Most questions focus on diagnosis or treatment and are aligned with clinical reasoning [2]. It is essential to consider how much information to convey to the audience before reaching the 1 or 2 key discussion points. For example, if the goal is to discuss the causative organisms of a brain abscess found on a computed tomography scan in a patient with fever and headache, posing the case for discussion by presenting only the information about the fever and headache before the computed tomography might lead the audience to discuss other diagnoses, such as meningitis or sinusitis, and the discussion about the causative organisms of the brain abscess may never be reached. The presenter is responsible for guiding the audience smoothly to the primary discussion points within a limited time. By identifying the key learning objectives or areas for discussion about the case, trainees can present corresponding clinical data and pose specific discussion questions to focus the audience. For example, instead of asking the audience about its next steps in managing the patient's brain abscess, the presenter could ask the audience how it would select an empiric antimicrobial regimen based on the spectrum of activity and the ability of the antimicrobial to penetrate the central nervous system. It is also important to specify which level of participants, such as residents, fellows, or faculty, will answer questions or provide differential diagnoses, rather than allowing open responses from the audience. In virtual settings, engagement can be enhanced through chat boxes, reaction buttons, or inviting attendees to unmute to encourage interaction.

### Present a Summary Slide for Audience Discussion

A summary slide about the patient's case can be highly useful during the discussion phase of a presentation. This summary, sometimes referred to as the “one-liner” or the problem representation, should concisely describe the key features of the case, such as pertinent demographic features of the patient (eg, immune status, relevant comorbidities), the time course of the illness (eg, acute, subacute, chronic), and the core syndrome (eg, brain abscess, meningoencephalitis, hospital-associated pneumonia). Sometimes, the presenter can ask an audience member to present one’s own problem representation, allowing early learners an opportunity to practice synthesizing a case or providing an expert an opportunity to demonstrate clinical reasoning and an ability to select key features of the case. Additionally, a well-structured summary slide can assist participants who join late or miss parts of the case, enabling them to reengage without revisiting all previously presented data. This not only lets the audience recap the case and engage in discussion but also helps presenters build their skills in clearly summarizing key clinical features, including pertinent positive and negative findings.

### Structure Teaching With Focused and Concise Learning Objectives

Clear learning objectives help presenters focus their message and enhance audience learning. Although not always required, many presentations include a brief didactic segment. Regardless of the format or timing, it is important to ensure that the scope of teaching points remains focused and concise. Starting with these points helps to orient the audience and decrease its cognitive load by providing a scaffold or framework of the information that the presenter will teach. A common pitfall is trying to comprehensively review all aspects of a disease or microorganism (eg, epidemiology, pathogenesis, clinical presentation, diagnosis, treatment, and prevention). Given the time constraints of conference settings, a more effective approach is to focus on the most relevant 2 to 3 points directly related to the case. Presenters should prioritize these teaching points that offer concrete and actionable takeaways—such as a diagnostic schema, management script element, or a common clinical pitfall—as these are often more memorable and impactful than broad overviews.

### Include Relevant Citations on Each Slide

One of the primary purposes of case conferences is to allow participants to virtually experience similar cases, motivate further learning, and enable them to apply this knowledge in diagnosing and treating future patients. Therefore, including references to data that support the main teaching points allows the audience to identify the primary source of the information presented and distinguish between the presenter's perspectives and published data. While some presenters list references at the end, including them at the bottom of each relevant slide is advisable so that the audience can correlate the information being presented with the reference. Some trainees will include a quick response (QR) code that links directly to the article to allow the audience easy access to the primary reference. This approach enhances the credibility of the presented data. Importantly, the process of conducting a literature search can itself be part of the learning objectives of a case presentation. Programs could offer guidance on how to conduct a high-quality literature review and identify which types of literature best support the educational goals of a case presentation, especially for presenters with less experience. Furthermore, during the teaching session, presenters can introduce practically useful review articles or guidelines to support learners' further learning.

### Use Visuals Wisely

Effectively utilizing charts, graphs, and images can significantly enhance and complement the narrative, making complex information easier to understand, especially in time-limited presentations. Visual aids should be relevant and present key points [7].

In ID case conferences, visual tools should be used to enhance clinical reasoning, clarify timelines, and highlight key findings. Examples include timeline graphics to illustrate the chronology of illness, hospital events, and antimicrobial treatment; standardized fishbones to display laboratory values; selected imaging studies with abbreviated reads; and microbiologic visuals such as plates, slides, or smears. Trainees may also consider creating simple tables to compare differential diagnoses or syndromes, which can clarify key distinctions more effectively than verbal explanations alone.

Slide design plays a role in clarity. The presenter can ensure readability by choosing appropriate font sizes, template designs, and color schemes [8]. Professional fonts such as Arial, Calibri, or Verdana are recommended, while fonts such Comic Sans or Papyrus, which may appear unprofessional, should be avoided. High-contrast colors, such as black on white or white on dark backgrounds, enhance readability. Red-green and yellow-white combinations should be avoided, as they may be difficult to distinguish, particularly for individuals with color vision deficiencies [8].

It is essential to aim to avoid overcrowding the slides with excessive detail as it can overwhelm the audience and detract from the main messages. If a busy table or figure from a reference is reproduced in the presentation, presenters can use boxes, highlights, or bolding to draw focus and attention to the key aspects of the data. Checking the size of visuals on a projected screen ensures clarity, preventing the need to apologize for unreadable slides. Like infographics and visual abstracts in research and education [9, 10], incorporating visual elements into presentation slides helps the audience better understand the teaching points. This approach aligns with Mayer's cognitive theory of multimedia learning, which underscores the value of combining words and visuals to enhance comprehension and retention [11]. Valuable tools for creating impactful presentations are suggested (Table 2). Trainees can learn how to use these tools through institutional educational programs or online resources such as Coursera [12], LinkedIn Learning [13], and Udemy [14]. Artificial intelligence tools can also assist in drafting tables or figures, though users must carefully verify accuracy before inclusion.

**Table 2.** Useful Tools for Case Presentation

Category: Tool/Resource	URL Link	QR Code
Platform		
Microsoft PowerPoint	https://www.microsoft.com/en-us/microsoft-365/powerpoint	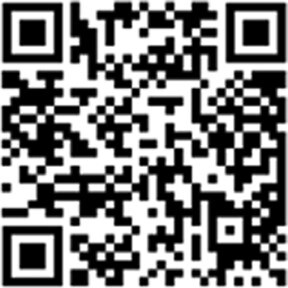
Google Slides	https://workspace.google.com/products/slides/	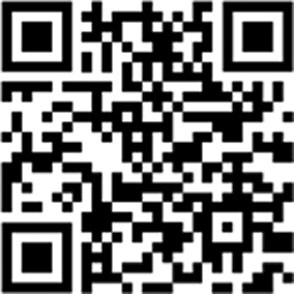
Canva	https://www.canva.com/	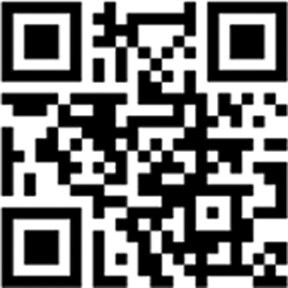
Beautiful. ai	https://www.beautiful.ai/presentation-maker	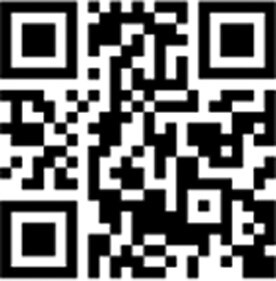
Prezi	https://prezi.com/	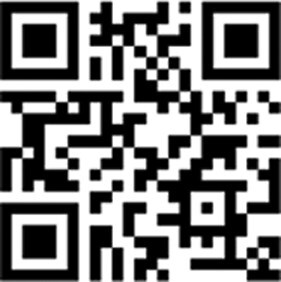
Icon, pictogram, and image creation		
Flaticon	https://www.flaticon.com/	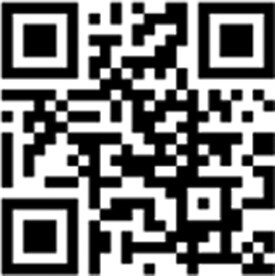
The Noun Project	https://thenounproject.com/	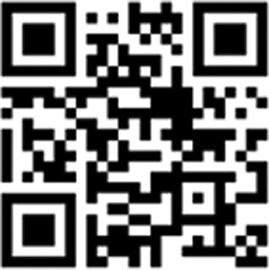
Icons8	https://icons8.com/	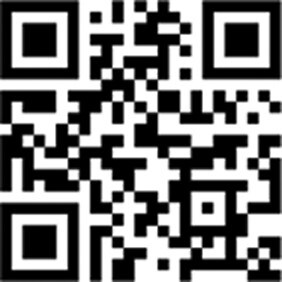
Freepik	https://www.freepik.com/	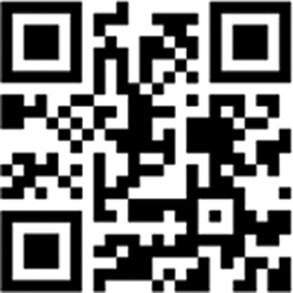
Iconfinder	https://www.iconfinder.com/	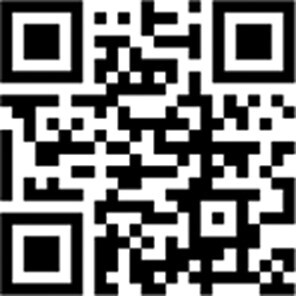
Piktochart	https://piktochart.com/	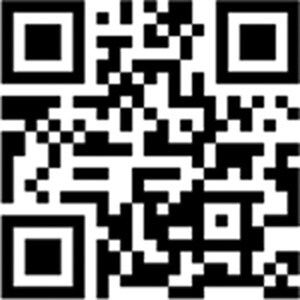
Adobe Express	https://www.adobe.com/express/	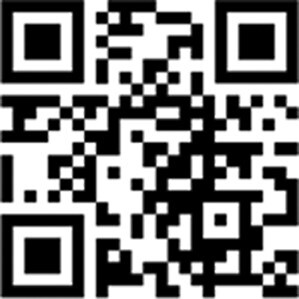
Bioicons	https://bioicons.com/	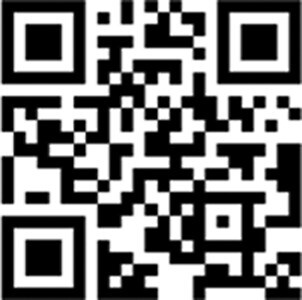
BioRender	https://www.biorender.com/	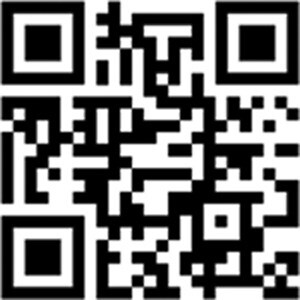
Engagement/poll maker		
Poll Everywhere	https://www.polleverywhere.com/	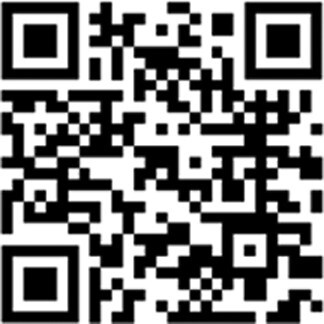
Slido	https://www.slido.com/	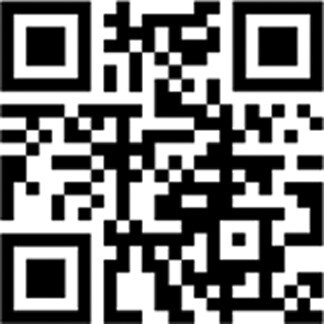
Vevox	https://www.vevox.com/	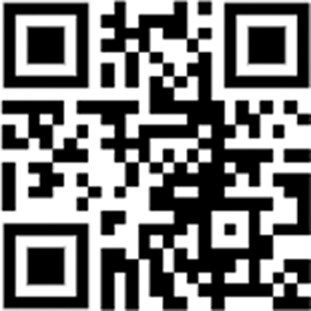
Mentimeter	https://www.mentimeter.com/	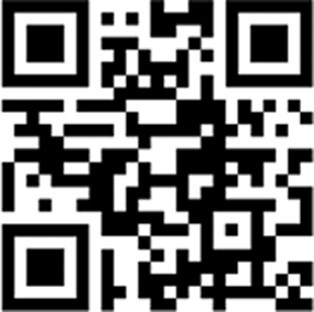
QR code generator		
QR Code Generator	https://www.qr-code-generator.com/	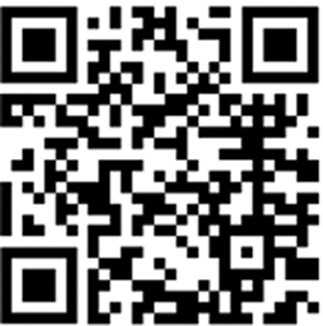
QRStuff	https://www.qrstuff.com/	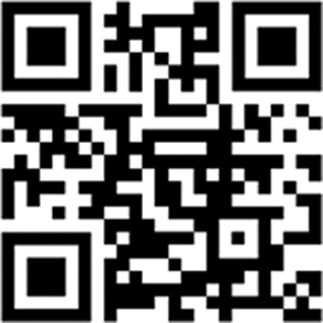
QR.io	https://qr.io/	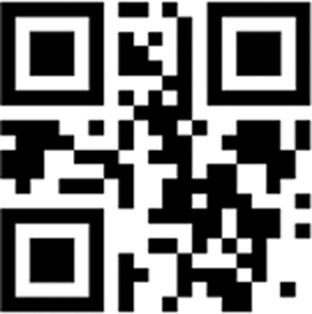

Abbreviation: QR, quick response.

### Summarize Key Points With Short Take-home Messages

Condensing presentations into short take-home messages is essential for maintaining focus and ensuring that the audience retains key points. In ID clinical case conferences, effective take-home messages often highlight diagnostic frameworks, common mimickers, atypical presentations, antimicrobial treatment strategies, or frequently encountered clinical pitfalls. These points should be practical, case specific, and clearly aligned with the presentation's learning objective. Guided by cognitive load theory, this approach reduces cognitive demands, enhancing the audience's ability to process and recall essential information [15]. A prospective randomized controlled study in France showed that including take-home messages on a slide significantly improved learners' retention [16]. However, the goal is to include only a few. Lautrette et al reported that two-thirds of participating young doctors could identify at most only 1 of the 3 main take-home messages from a lecture [17]. While the number of take-home messages is a topic of debate, limiting them to 3 or 4 is considered reasonable [5, 17, 18].

### Manage Time Effectively

Rehearsing the presentation to ensure that it fits within the allotted time is crucial for a successful delivery. Thorough preparation also helps avoid common pitfalls, such as technical issues with screen sharing or microphone problems, which are especially common in virtual or hybrid conferences. This is particularly important in ID case conferences, where presentations are often followed by multidisciplinary discussions and cases may involve complex diagnostic timelines, microbiologic data, or antimicrobial treatment courses—all of which can consume significant presentation time if not carefully structured. Some presenters attempt to cover too many topics, leading to lengthy talks that can distract the audience. Respecting the participants' time by finishing within the scheduled time frame is important. Concluding promptly allows sufficient time for feedback and questions [5]. It is challenging for the audience to stay focused if the presentation extends beyond the scheduled end time. The presenter loses effectiveness when rushed to finish the prepared material before time runs out.

### Share the Slides and Seek Feedback

At the end of the presentation, providing the audience with access to presentation materials is highly encouraged. Some institutions archive presentations for future reference. Emailing the materials or using QR codes on the presentation slide for easy sharing can facilitate further learning for the audience. The presenter should maintain patient confidentiality by removing identifiable personal information, sharing only the educational content, and gathering feedback after the presentation. The presenter can collect feedback through mentors' observations or surveys shared via email or QR codes included on the last slide. This can also be placed on the wall or doors prior to people leaving. This input helps presenters identify areas for improvement and refine their content and teaching effectiveness [19]. In addition to slide sharing, presenters may consider repurposing educational components of their presentation—such as diagnostic schemas, short clinical vignettes illustrating key decision points, or simplified summary of antimicrobials—as reusable asynchronous learning or teaching tools for rounds. These materials can be shared as handouts, posted on learning platforms, or incorporated into institutional shared drives to extend the educational value of the case conference beyond the session itself.

## CONCLUSION AND FUTURE DIRECTION

These 10 practical tips offer a roadmap for trainees to navigate the challenges of case presentations, enhancing the quality of their presentations and the learning experience for their audience. The presentation preparation checklist is outlined in Table 3. It is necessary to evaluate the impact of these presentation tips on trainee performance and learning outcomes. Educational research could explore the role of structured interventions, such as workshops, quality improvement, or mentoring programs, in enhancing trainees' case presentation skills within the context of ID case conferences during their training. Additionally, incorporating feedback into case conferences could help identify areas for further improvement. Ultimately, fostering a culture of continuous improvement in presentation skills will benefit trainees and enhance the overall quality of education in the ID field and patient care.

**Table 3.** Presentation Preparation Checklist for Infectious Disease Clinical Case Conferences

Tip		Checklist Items
1	Choose educationally relevant cases and topics	Select a case with educational messages. Ensure that the topic has not been presented recently.
2	Include pertinent positives and negatives	Identify and include significant positive and negative findings. Exclude irrelevant details to keep the presentation focused.
3	Consider the points for discussion	Choose 1 or 2 key points of the case for focused discussion. Plan where to pause the presentation for audience engagement.
4	Prepare a summary slide for audience discussion	Create a summary slide with key findings for the discussion.
5	Structure teaching with focused and concise learning objectives	Clearly define the main educational points for the audience. Avoid a general overview; focus on what can be learned from the presented case.
6	Include relevant citations on each slide	Attach relevant citations to each slide. Distinguish between your perspective and evidence-based information.
7	Use visuals wisely	Choose appropriate font sizes, design templates, and color schemes. Incorporate relevant charts, graphs, or images. Avoid overcrowding slides with excessive detail.
8	Summarize key points with short take-home messages	Conclude with a summary slide containing 3 or 4 key take-home messages. Keep messages concise.
9	Manage time effectively	Rehearse your presentation to ensure that it fits within the allotted time. Prepare for potential technical issues. Conclude promptly, leaving enough time for feedback and questions. Avoid extending beyond the scheduled end time.
10	Share the slides and seek feedback	Offer slides via email, quick response code, or institutional archive. Ensure that patient confidentiality is maintained. Collect feedback for improvement.
